# Effects of Stannous Fluoride Toothpaste and Chios Mastiha Toothpaste on the Prevention of Enamel Erosion

**DOI:** 10.3290/j.ohpd.c_2301

**Published:** 2025-10-07

**Authors:** Konstantina Chatzidimitriou, Spyros Papageorgiou, Sotiria Gizani, William Papaioannou

**Affiliations:** a Konstantina Chatzidimitriou Department of Preventive & Community Dentistry, National and Kapodistrian University of Athens, School of Dentistry, 2 Thivon Str, 115 27, Goudi, Athens, Greece. Substantially contributed to conception and design, to acquisition, analysis and interpretation of data, drafted and critically revised the manuscript for important intellectual content and gave final approval.; b Spyros Papageorgiou Department of Biomedical Sciences, Laboratory of Chemistry-Biochemistry-Cosmetic Science, University of West Attica, Panepistimioupolis Egaleo Park, 12243 Athens, Greece. Substantially contributed to conception and design, to acquisition, analysis and interpretation of data, critically revised the manuscript for important intellectual content and gave final approval.; c Sotiria Gizani Department of Paediatric Dentistry, National and Kapodistrian University of Athens, School of Dentistry, 2 Thivon Str, 115 27, Goudi, Athens, Greece. Substantially contributed to conception and design, to acquisition, analysis and interpretation of data, critically revised the manuscript for important intellectual content and gave final approval.; d William Papaioannou Department of Preventive & Community Dentistry, National and Kapodistrian University of Athens, School of Dentistry, 2 Thivon Str, 115 27, Goudi, Athens, Greece. Substantially contributed to conception and design, to acquisition, analysis and interpretation of data, critically revised the manuscript for important intellectual content and gave final approval.

**Keywords:** enamel erosion, salivary pellicle, dental protection, toothpaste, stannous fluoride, mastic

## Abstract

**Purpose:**

Erosive tooth wear (ETW) is an increasingly prevalent condition characterised by the loss of dental hard tissue due to repeated effects and interactions of acids and mechanical forces. The aim of this study was to investigate the primary preventive effect of salivary pellicle combined with either stannous fluoride (SnF_2_) toothpaste or mastic toothpaste against ETW in permanent teeth.

**Materials and Methods:**

Using a three-arm *invitro* model, 54 enamel samples (18 in each group) were prepared with a salivary pellicle alone (control), salivary pellicle with SnF_2_ toothpaste, or salivary pellicle with mastic toothpaste. The experimental design consisted of 5 cycles of salivary pellicle formation (30 min, 37°C), modification with the solutions (30 min, 25°C), further salivary pellicle formation (2 h, 37°C) and erosive challenge (2 min, 1% citric acid, pH 3.6). Subsequently, the samples were evaluated by optical profilometry, Vickers hardness (VH), and scanning electron microscopy coupled with energy-dispersive X-ray spectroscopy (SEM/EDX).

**Results:**

SnF₂ group exhibited significantly reduced surface roughness (Sa, Sq, Sc, Sm, Sv parameters) compared to other groups and lower total hardness loss (ΔVH: SnF_2_ 23%, control 26%, mastic 36%) than the mastic group. SEM analysis revealed better preservation of the prismatic enamel structure in the SnF^2^ group, indicating resistance to acid-induced demineralisation. Mastic toothpaste did not provide significant protection against erosion, raising questions about its suitability for preventing ETW in acidic environments. EDX analysis showed no significant differences in elemental composition among the groups.

**Conclusions:**

This study confirmed the use of SnF_2_ as an effective agent for protecting enamel against ETW. While natural extracts like mastic may have antimicrobial benefits, their protective role against ETW appears limited, emphasising the need for further research to explore their promising potential applications in oral prevention as well as their limitations. The future of ETW prevention may lie in the synergy between natural and synthetic agents, combining efficacy with biocompatibility and patient acceptance.

Erosive tooth wear (ETW) is defined as the loss of hard dental tissue resulting from the repeated effects and interactions of acids and mechanical forces.^26^ In recent years, both the frequency and severity of ETW are steadily increasing in children and adolescents, while researchers estimate that 30–50% of primary teeth and 20–45% of permanent teeth are affected by ETW.^37^ The etiological factors of ETW are both dietary and patient related. Among dietary factors, the consumption of soft and energy drinks is the most significant external contributor,^27^ while among patient-related factors, the most significant are gastroesophageal reflux disease and eating disorders.^14^

At the tooth level, it is well known that depending on the pH of the erosive agent, short erosion times of up to 3 min might result in a softened enamel layer of approximately 0.5 μm, which is prone to brushing wear.^45^ One of the most critical biological defences against this softening of the enamel is saliva, and specifically the salivary pellicle. This dense basal layer, primarily consisting of mucins, histatins, statherins, and proline-rich proteins,^27,46^ contributes to the protective action against demineralisation, enhancing resistance to acids.^15^ The thicker the salivary pellicle, the greater the protection of the tooth surface.^46^ For this reason, efforts have been made in the literature to enhance the protective effect of the salivary pellicle against ETW.

Despite growing awareness of ETW and its contributing factors, preventive measures still remain limited and unevenly studied in the literature.^10^ Regarding the use of fluoride products, conventional products with low and medium fluoride concentrations and neutral pH, have limited preventive action. However, fluoride formulations containing polyvalent metals show the most promising results, particularly those combining stannous and fluoride (SnF_2_).^26^ The application of these formulations promotes the formation of a metal-rich surface layer with increased fluoride uptake and higher resistance to acids.^18^

However, both parental concerns regarding potential toxicity from increased ingestion of fluoride-containing products and the notable propensity of SnF_2_ to cause extrinsic stains have led to a shift towards alternative options, such as certain natural extracts.^24,29^ Some plant extracts have been shown to increase the thickness of the salivary pellicle.^47^ This effect is associated with the polyphenol content of the extracts, as polyphenols can aggregate salivary proteins, increasing protein adsorption on the enamel surface, thereby enhancing the protective effect of the salivary pellicle against ETW. One particularly intriguing natural product is *Chios Mastiha* (mastic), a natural, aromatic resin obtained from the mastic tree (*Pistacia lentiscus L. var latifolius Coss* or *Pistacia lentiscus var. Chia*), which is exclusively produced in the Greek island of Chios. Mastic derivatives are complex mixtures of various phytochemical groups, including phenolic compounds, many of which are closely linked to their antimicrobial, antioxidant, anti-inflammatory, and anticancer properties. Concerning their effects on the oral cavity, they have been found to offer additional antibacterial action against *Streptococcus mutans*, thereby contributing to the prevention of caries.^1,30^ However, despite the promising properties of mastic, a significant knowledge gap exists in the literature regarding its role in preventing ETW. Mastic’s natural composition, combined with its ability to enhance the salivary pellicle, positions it as a compelling alternative or complement to conventional fluoride-based approaches, offering a potential breakthrough in ETW prevention strategies. To date, no studies have been conducted linking mastic to the prevention of ETW. This lack of research underscores the need to explore Mastic’s potential in addressing a growing oral health concern.^38^

Therefore, the aim of this *invitro* study was to investigate in permanent teeth the primary preventive effect of the combination of salivary pellicle with SnF_2_ or mastic toothpaste against ETW. The null hypothesis was that the combination of salivary pellicle with SnF_2_ or mastic toothpaste will not provide any protective effect against ETW of permanent teeth.

## MATERIALS AND METHODS

### Ethics

The present experiment was carried out in accordance with the approved regulations of the Ethics committee of the School of Dentistry, National and Kapodistrian University of Athens (NKUA) (489/20.01.2022). Human saliva was collected from a healthy donor, and informed consent was obtained. Oral consent was also obtained from patients who donated teeth, which were extracted in the postgraduate clinic of the Department of Paediatric Dentistry, School of Dentistry (NKUA).^11^

### Sample Size Calculation

The sample size calculation was based on the difference in calculated surface loss for different toothpaste treatments from a previous study^13^ and considered a balanced one-way analysis of variance power calculation (f = 0.4, level of significance = 0.05, 80% power); it indicated a minimum number of 18 in each group and therefore 54 sound premolars were finally collected.

### Sample Preparation

Caries-free premolars were stored in a 0.1% thymol solution after their extraction. The crowns were separated from the roots using an Isomet 110 Low Speed Saw (Lake Bluff, IL, USA), and then the labial surfaces were embedded in resin (Paladur; Heraeus Kulzer, Hanau, Germany) in two parallel moulds. The teeth in the thicker mould (7.5 mm thick) were serially abraded after the thinner mould (200 μm thick) was removed. Using water-cooled silicon carbide paper disks (grain size 400, 1,000, 2,400, 4,000), the specimens were serially ground (Dap-V, Struers, Ballerup, Denmark). The samples were then polished for 3 minutes using a 3 μm diamond paste (DP-3, Struers). A standardised 200 μm layer of outer enamel was removed throughout this process, leaving samples polished and flat. A layer of colored nail varnish was applied to a portion of the exposed enamel area for later determination of intact and treated tooth surface.

### Groups

The specimens were divided into three groups: (1) salivary pellicle (control); (2) salivary pellicle and SnF_2_ toothpaste; (3) salivary pellicle and mastic toothpaste. 

### Collection of Stimulated Human Saliva

Stimulated human saliva was collected from a single healthy donor aged 30 years, who was informed not to eat or drink for 2 h before the saliva collection, performed between 9:00 a.m. and 10:00 a.m. The donor chewed on paraffin wax for 10 min, and all stimulated saliva was collected in chilled vials. The saliva was then pooled and centrifuged for 20 min at 4°C (4,000 g). After separating the supernatants, the aliquoted saliva was stored in 2.5 mL aliquots at –80 °C until the experiment day.^31^

### Toothpaste Slurries Preparation

Two different toothpastes were tested in this study (Table 1). 5 g +/–0.01 g of each toothpaste with SnF_2_ and mastic toothpaste were accurately weighed in a separate 50 mL beaker, and 10 mL of deionised water was added to it. The mixture was initially agitated with a glass rod and then homogenised with a magnetic agitator for 15 min at 200 rpm and 250°C, until a fully slurry toothpaste was achieved. Then, the pH was measured directly in the slurry solution of toothpastes and adjusted if needed to neutral (pH = 7.0 +/–0.2).

**Table 1:** Description of groups and toothpaste ingredients used in the study

Group	Toothpaste composition (INCI Names)	Ingredient of interest	Brand/manufacturer
2: Salivary pellicle and SnF_2_ Toothpaste	Glycerin, hydrated silica, sodium hexametaphosphate, PEG-6, propylene glycol, aqua, zinc lactate, trisodium phosphate, sodium gluconate, sodium lauryl sulphate, aroma, sodium saccharin, CI 77891, stannous fluoride, stannous chloride, xanthan gum, PVP, carrageenan, sodium fluoride, sodium hydroxide	SnF_2_ (1,100 ppm F-), NaF (350 ppm F-)	Oral-B gum and enamel repair Original® toothpaste
3: Salivary pellicle and Chios Mastic toothpaste	Sorbitol, pistacia lentiscus (mastic) gum water, glycerin, hydrated silica, PEG-32 PVM/MA copolymer, pistacia lentiscus (chios mastiha) powder, pistacia lentiscus (chios mastiha) oil, titanium dioxide, sodium lauroyl sarcosinate, xanthan gum, sodium benzoate, sodium hydroxide, sodium saccharin	Mastic water 15.8% w/w, mastic oil 0.21% mastic dry extract 1% w/w	Experimental formulation developed by the Laboratory of Chemistry-Biochemistry- Cosmetic Science, Department of Biomedical Sciences, University of West Attica


### Experimental Procedures

For pellicle formation, one aliquot was taken from the freezer and thawed at 37°C. In the control group, each enamel specimen received an aliquot of 160 μl of saliva, and the enamel specimens were stored in a humid chamber at 37°C for 2 h. In groups 2 and 3, salivary pellicle formation (30 min, 37°C, no agitation), followed by pellicle modification with the experimental solutions (30 min, 25°C, 70 rpm, travel path 50 mm), and subsequent salivary pellicle formation (2 h, 37°C, no agitation) were performed.^29^ The specimens were then submitted to an erosive challenge (2 min, 1% citric acid, pH 3.6, 70 rpm, travel path 50 mm). After each procedure, the specimens were rinsed with deionised water (20 s), dried with air (5 s) and then kept in a humid chamber overnight, at room temperature, until the next experimental cycle consisting of pellicle formation and erosion.^4,13^ A total of five experimental cycles were carried out.

### Optical Profilometry

After the erosion cycles, the colored nail varnish was carefully removed with a scalpel, thus exposing the intact enamel layer. The surface roughness parameters of each group were identified by an optical interferometric profiler (Wyko NT1100, Veeco, Tucson, AZ, USA). Profilometric readings and measurements were performed on the intact, treated, and interfacial regions of the specimens (n = 3). A Gaussian regression filter was applied with a 0.0025 mm short-wavelength cut-off filter (high-pass), and then the surface roughness parameters presented in Table 2 were recorded. All acquired data were treated employing the Vision 64, ver 5.7 software (Bruker Corporation, Tucson, AZ, USA).

**Table 2:** Symbols, definitions and interpretations of selected surface roughness parameters

Parameter	Definition	Interpretation
Sa	Arithmetic average height of the surface	An overall measure of the surface texture but insensitive to various texture characteristics.
Sq	Root mean square height of the surface
Sz	Average difference between the five highest peaks and five lowest valleys	Characterization of the maximum peak to valley magnitude for the entire surface and demonstration of a change sooner than Sa or Sq.
Ssk	Skewness of height distribution	Ssk >0 The surface is characterized by peaks. Ssk <0: The surface is dominated by valley.
Sku	Kurtosis of height distribution	Surfaces composed of inordinately high peaks/deep valleys have Sku >3.00. Sku <3.00 indicates the absence of inordinately high peaks/deep valleys.
Sc	Core void volume	The volume that the surface would support from 10–80% of the bearing ratio.
Sm	Surface material volume	The amount of material contained in the surface peaks from 0% to 10% of the bearing area ratio.
Sv	Surface void volume	The volume (for example, of a fluid filling the valleys) that the surface would support from 80% to 100% of the bearing ratio.
Sdr	The percentage of additional surface area contributed by the texture as compared to an ideal plane the size of the measurement region	Differentiation of surfaces of similar amplitudes and average roughness. It increases with the spatial intricacy of the texture, whether or not Sa changes.
Sds	Summit density	The number of summits per unit area that make up the surface.


### Vickers Hardness (VH)

All specimens were subjected to VH testing using a hardness tester machine (ΗΜ 2000, Shimadzu, Kyoto, Japan) at ambient temperature (ISO 6507-1:2018). A maximum load force of 50 g was applied, and the dwell time was set at 10 s. Three indentations per specimen were carried out at the intact and treated regions, and their mean hardness value was considered as the hardness value for the specimen. The percentage difference in VH (ΔVH) was calculated using the formula ΔVH = VHint – VHtr / VHint × 100, where VHint was the VH in the intact region and VHtr was the value in the treated region after the last erosion cycle.

### Scanning Electron Microscopy and X-ray Energy-Dispersive Spectroscopy (SEM/EDX)

Four samples from each group were randomly selected, sputter-coated with carbon (Baltec SCD 004) and taken to the SEM/EDX chamber (Quanta 200, FEI, Hillsboro, OR, USA). SEM images of the intact and treated enamel surfaces were taken at 1,200× nominal magnifications, with an acceleration voltage of 20 kV and 97 μA beam current.

Then, the elemental composition of intact and treated areas was determined by EDX employing a silicon drift detector (X Flash 6|10, Bruker, Berlin, Germany) with a slew-window under the same accelerating voltage and beam current and a 110 × 110 μm acquiring window. The spectra were acquired and quantified using dedicated software (ESPRIT ver. 1.9, Bruker, Berlin, Germany), which employed ZAF (atomic number – absorbance – fluorescence) and carbon coating correction routines. Carbon was excluded from the quantification due to its presence in the conductive coating.

### Statistical Analysis

The Shapiro–Wilk test was used to evaluate the normality of distribution of the parameter values at baseline and after erosive challenge and of the relative parameter alterations among the different groups to determine whether a parametric or a non-parametric analysis should be performed in each case. Kruskal–Wallis one-way analysis of variance on Ranks was conducted to examine if there was a statistically significant difference among groups. In case of significant results, Dunn pairwise tests were carried out to compare all pairs of groups. The level of statistical significance for all the analyses was set at 0.05 (a = 0.05). Statistical analyses were performed using IBM SPSS Statistics 22 software (IBM).

## RESULTS

### Surface Roughness

**Fig 1:**
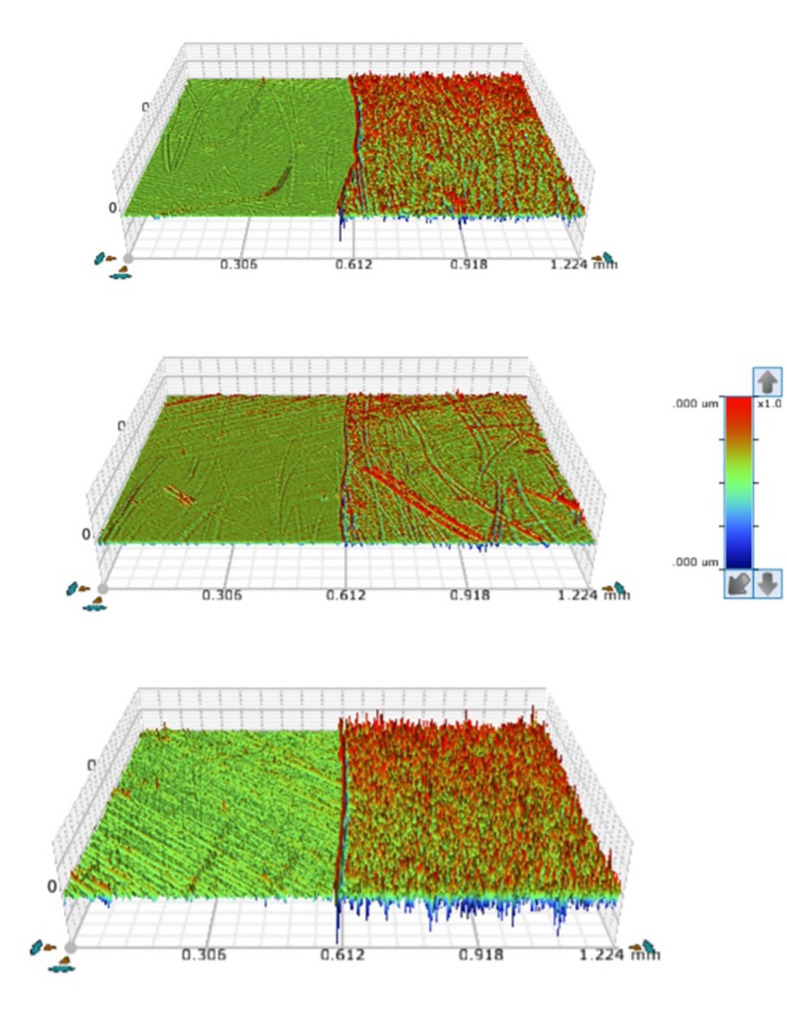
Representative 3D optical profilometric images of interfacial regions from all groups tested. (a) Control; (b) SnF_2_; (c) mastic.

Figure 1 demonstrates representative 3D optical profilometric images of interfacial regions from all groups tested. Kruskal–Wallis one-way analysis of variance on ranks and Dunn’s test demonstrated statistically significant differences among groups for the Sa, Sq, Sc, Sdr, and Sds parameters (P < 0.001). More specifically, SnF_2_ group showed statistically significant (P < 0.001) lower values [Sa = 33 (28,44); Sq = 49 (41,66); Sc = 46 (41,66); Sdr = 0.0 (0.0, 0.1); Sds = 0.003 (0.002, 0.003)] than the control [Sa = 75(63,98); Sq = 102 (89, 129); Sc = 108 (90, 141); Sdr = 0.2 (0.1, 0.3); Sds = 0.004 (0.004, 0.004)] and mastic group [Sa = 83 (72, 99); Sq = 116 (98, 133); Sc = 120 (108, 147); Sdr = 0.3 (0.2, 0.4); Sds = 0.004 (0.004, 0.004)]. However, no significant differences were found between the control and the mastic group. Regarding the Sz parameter, Kruskal–Wallis one-way analysis of variance on ranks showed no significant differences between groups [Control, Sz = 2265 (2008, 3281); SnF_2_, Sz = 2173 (1374, 2418); mastic, Sz = 2139 (1888, 2859)]. For the Sku parameter (kyrtosis), it was shown that all values were greater than 3.00, demonstrating surface composition of inordinately high peaks/deep valleys in all groups [SnF_2_ group = 14 (10, 19); control group = 6 (5, 7); mastic group = 5 (5, 6)]. For the Ssk parameter, the values for all groups were equal to 0, indicating symmetry of the surface heights about the mean plane and no preponderance of peaks (Ssk > 0) or valley structures (Ssk < 0) comprising the surface. For parameters Sm and Sv, Tukey test indicated that SnF_2_ group showed statistically significant (P <0.001) lower values [Sm = 4(2); Sv = 7(3)] than the control [Sm = 7(2); Sv = 13(2)] and mastic group [Sm = 8(2); Sv = 14(3)]. No significant differences were found between the control and the mastic group.

### Vickers Hardness (VH)

**Fig 2:**
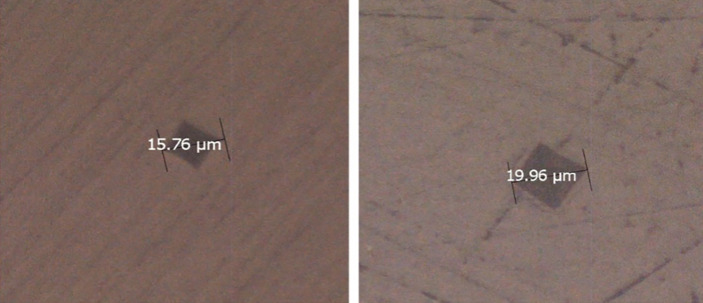
Pyramid-shaped Vickers indentation marks on enamel sample of intact (a) and treated after five erosion cycles (b) enamel surfaces in the mastic group.

The median values of the total hardness loss, along with interquartile range (IQR), were 26% (11, 40) for control, 23% (13, 29) for SnF_2_ and 36% (26, 41) for the mastic group. Kruskal–Wallis one-way analysis of variance on ranks and Dunn’s test showed statistically significant (P < 0.05) lower values of total hardness loss for SnF_2_ than the mastic group. No significant differences were found between the control and mastic groups and between the control and SnF_2_ group (Fig 2).

### SEM/EDX

**Fig 3:**
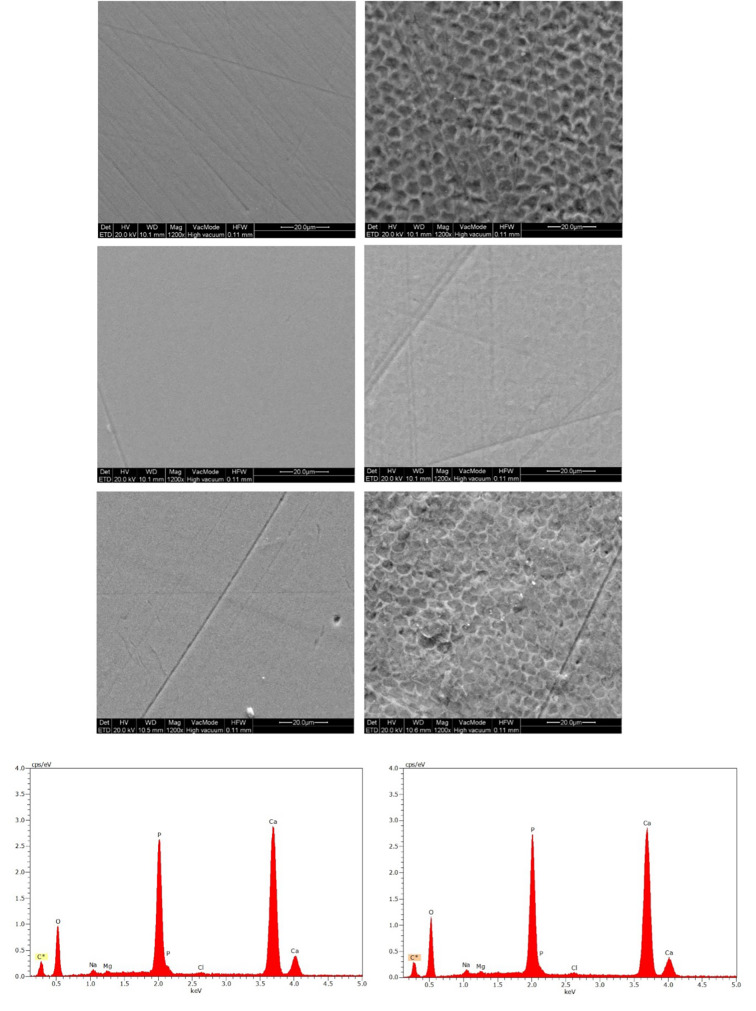
SEM images of intact (left column of images) and treated after five erosion cycles (right image column) enamel surfaces in control (a), SnF_2_ (b) and mastic (c) groups. EDX spectra for enamel samples of intact (left column of images) and treated after five erosion cycles (right column of images) surfaces.

On all SEM images, the effect of erosion cycles on the enamel surface is clearly visible, with a distinct, demineralised prismatic surface. SnF_2_ toothpaste was better able to preserve the prismatic structure, with the enamel surface in the SnF_2_ group showing fewer signs of erosive demineralisation. No distinguishing characteristics could be observed between the control and mastic groups (Fig 3).

The elements identified were O, Na, P, Mg, Cl, and Ca. The EDX elemental weight percentage recorded for the intact and treated enamel surfaces and the integration of peaks corresponding to various elements presented similar results in all groups. All EDX spectra also showed no Sn on the enamel surfaces (Fig 3).

## DISCUSSION

This study investigated the primary preventive effects of salivary pellicle combined with either SnF_2_ or mastic toothpaste against ETW in an invitro model. The findings provided important insights into the protective potential of these interventions, contributing to the growing body of evidence addressing the prevention of ETW in permanent teeth. The null hypothesis that there would be no differences among the different groups was rejected for changes in VH and surface roughness but was accepted for changes in EDX analysis.

The study reinforced the well-established protective effects of SnF₂ in preserving enamel integrity against erosive challenges,^36^ as Sn_2_OHPO_4_, Sn_3_F_3_PO_3_, and Ca(SnF_3_)_2_ salts can be formed when Sn^2+^ ions react with the dental hard tissue, creating a stable and acid-resistant layer on the tooth surface.^5^ O’Toole et al (2015) showed that overall, compared to sodium fluoride, SnF_2_ caused statistically lower mean step heights when applied before the erosive challenge; this finding was in accordance with results of other studies, too.^17,21–23,51^ However, other authors found that SnF_2_ toothpaste removed more of the weakened enamel layer rather than creating a protective layer on the enamel surface.^2,25^

Despite its efficacy, SnF_2_ presents challenges, including potential extrinsic staining and parental concerns regarding fluoride toxicity. These limitations necessitate further research into developing alternative treatments to enhance application and acceptance in clinical practice. The present study’s protocol was based on the hypothesis that mastic polyphenols would attach to salivary pellicle proteins and attract additional ones, changing the salivary pellicle more effectively and enhancing its protective effect.^28,42^ In the literature, various studies have already been conducted regarding the protective effect of several natural extracts against ETW. Taking into account teas, the salivary pellicle’s protective function was enhanced by green and black tea, due to their high polyphenolic contents.^40,47^ According to Niemeyer et al,^29^ grape seed and grapefruit seed extracts presented the most promising results due to their major components, attracting additional proteins to the basal layer of the pellicle and ultimately altering its structure and improving its protective effect.^7,33,52^ However, the low pH of cranberry extract negated any potential beneficial effects and led to greater demineralisation.^24^ In our study, the pH of mastic toothpaste was 7 +/–0.2, and even after the long period of immersion, pH could not be responsible for the study results. The lipophilicity of terpenes and terpenoids contained in mastic oil and powder, combined with the difficulty in dispersing the mastic powder, and the fact that the product is exfoliating due to the size of the mastic powder particles, were some negative factors that could significantly impact the results. Moreover, mastic water, which is the upper water-soluble fraction of mastic resin, has been proven to be less effective due to the very low content of active ingredients compared to mastic oil and dry extract. Furthermore, Stannous and Sodium Fluoride are water-soluble and may attach better in the salivary pellicle than oil-soluble masticatory actives. Despite these limitations, mastic’s antimicrobial and antioxidant properties remain well-documented, making it a valuable candidate for oral health applications. The current findings highlight the need for innovation in mastic toothpaste formulations to unlock its potential in ETW prevention. Possibly, other mastic derivatives entrapped in water-soluble vehicles or delivery systems with prolonged release or other lyophilised mastic derivatives with improved water solubility and smaller particle size, may be produced and tested in the future.

In this study, several surface roughness parameters were analysed to assess the impact of SnF_2_ and mastic toothpaste on enamel surfaces. Specifically, Sa and Sq values in the SnF_2_ group were approximately half those of the control and mastic groups, signifying that SnF_2_ was more effective in maintaining the initial enamel surface pattern following erosive challenge. These findings are consistent with the reported protective effect of SnF₂ against acidic attacks on enamel in previous studies.^8,49^

Conversely, no significant differences were observed in the Sz parameter among groups, suggesting that while SnF₂ may reduce the overall surface roughness, it does not significantly alter the extreme peaks and valleys present on the enamel surface. This could indicate that SnF₂ acts more effectively in reducing surface irregularities rather than eliminating deeper erosive lesions. However, it must be considered that the Sz examination alone may not be instructive of the aggregate surface topography of a tested material,^16^ or it may also be vulnerable to excessive variability, measurement noise or contamination because of the reliance on maximal values.^34,35^

The kurtosis (Sku) values, greater than 3.00 for all groups, reflect a non-Gaussian distribution of surface heights with pronounced peaks and valleys in all treatments. This parameter indicates the presence of rough surface features in all groups, although SnF_2_-treated surfaces were comparatively smoother. Similarly, skewness (Ssk) values around 0 indicate symmetry in surface height distributions across all groups, suggesting that neither peaks nor valleys were excessively dominant, which aligns with the protective effects observed with SnF₂.

Functional parameters such as Sc, Sm, and Sv also showed that SnF_2_ contributed to a more compact and less porous enamel surface, possibly due to the formation of a protective layer that mitigates demineralisation.^18^ Hybrid (Sdr) and spatial parameters (Sds) also reinforced the effectiveness of SnF_2_ in preventing enamel roughness, as significantly lower values were observed in the SnF_2_ group.^3^ In contrast, mastic toothpaste did not offer significant protection against enamel roughness. This finding called into question the efficacy of mastic in ETW prevention and suggested that its use may be more beneficial for antimicrobial purposes rather than enamel protection against acidic environments.

Furthermore, in this study, SnF_2_ group exhibited a significantly lower percentage loss of VH compared to the mastic group after the erosive challenge. This finding reinforces the notion that SnF_2_ provided better protection against erosive softening of enamel, likely due to its ability to enhance the mineral density of the surface layer.^8^ The lack of significant differences between the control and SnF₂ groups may be attributed to the inherent variability in the enamel samples or the limitation of the hardness test in detecting subtle differences in protective effects. Nonetheless, the significantly higher VH loss in the mastic group suggested that mastic toothpaste offers minimal protection against enamel softening in comparison to SnF₂, which aligned with the surface roughness and SEM findings.

Similarly to our results, in the study of Carvalho and Lussi (2014), analysing the F/Sn/chitosan toothpaste group, it showed comparable total surface hardness loss as the NaF toothpaste group. This was likely due to the limited nature of surface hardness quantification.^32^ In other previous studies, within the first 10 min of erosion, rapid loss of enamel hardness was measured, followed by a stabilisation period where hardness values were nearly constant.^20,39^ It seems that if the conditions are mild enough, surface assessments like hardness or roughness may be more appropriate. But as the severity increases, the softened layer stabilises and the variations become imperceptible.^12^

Regarding the results of in situ studies, a recent meta-analysis comparing the effect of SnF_2_ toothpastes with control toothpastes for treatment of dentine hypersensitivity and enamel erosion, showed that SnF_2_ toothpastes provided an 83% benefit versus control toothpastes (P < 0.001) with a change (95% CI) in average surface profilometry level (μm) of –2.02 (–2.85, –1.20), confirming also at clinical settings the protective effect of SnF_2_ against ETW.^50^

SEM and EDX analyses provided morphological and elemental insights into the enamel surfaces before and after the erosive challenge. Although the secondary electron images only serve as illustrations, backscattered electron images revealed clear evidence of erosion in all groups. The SnF₂-treated group exhibited significantly less surface demineralisation and more intact tooth structure compared to the control and mastic groups, aligning with previous reports on the erosion-inhibiting effects of SnF_2_.^9,23,44^ The ability of SnF_2_ to preserve the prismatic structure of enamel could be attributed to its known effects in forming a stannous phosphate layer, which acts as a physical barrier against acid attacks.^6^

The EDX analysis revealed no significant differences in elemental composition between the intact and treated enamel surfaces across all groups. Notably, no detectable Sn was found on the enamel surfaces of the SnF_2_-treated group, which might suggest that the protective stannous layer is too thin to be detected by EDX or that the Sn ions may be incorporated into the enamel subsurface at a depth beyond the detection limits of the technique.^41^ In another study^13^ the EDX spectra from the surfaces of placebo and NaF toothpaste groups showed no Sn on the enamel surface. On the contrary, for the groups treated with the F/Sn/chitosan toothpaste, the EDX spectra revealed signals of Sn.^13^ This potential additional preventive effect of chitosan in the presence of Sn could be explained by the fact that, like chitosan, Sn can interact with salivary proteins,^19,43^ which in turn interact with the enamel. Therefore, it is plausible that the chitosan multilayer may also protect the Sn bonded to salivary proteins and enamel, enhancing the protective function of the protein, Sn, and chitosan complex structure.^13^

This study makes a valuable contribution to the literature on preventive dentistry, confirming the use of SnF₂ as an effective agent for protecting enamel from ETW. While natural extracts such as mastic may offer antimicrobial benefits, their protective role against ETW appears limited. Nonetheless, mastic remains a promising natural agent with future potential, provided that appropriate formulations are developed. The need for further research into the possible applications of natural substances is clearly highlighted, as the future of ETW prevention may lie in the synergy between natural and synthetic agents, combining efficacy with biocompatibility and patient acceptance.

## CONCLUSIONS

SnF_2_ toothpaste proved effective in creating a smoother, less porous enamel surface, reducing surface roughness compared to the control and mastic groups. SnF_2_ also provided better protection against enamel softening, with a lower percentage VH loss compared to mastic toothpaste.
Mastic toothpaste did not provide significant protection against erosion, raising questions about its suitability for preventing ETW in acidic environments. The development of more effective mastic toothpastes or other oral care mastic products against ETW may be achieved if the mastic oil, powder, or other derivatives are entrapped or lyophilised, increasing the water solubility, dispersion, delivery, and finally attachment and retention of mastic lipophilic active ingredients in enamel.
SEM revealed that SnF_2_-treated enamel surfaces displayed less demineralisation than the control and mastic groups. EDX analysis showed no significant differences in elemental composition among the groups.
The findings highlighted that while SnF_2_ still remains the gold standard for ETW prevention due to its robust enamel-protective properties, mastic toothpaste represents a natural alternative with potential for future development. Addressing the limitations identified in this study through advanced formulation techniques and rigorous clinical testing may pave the way for mastic derivatives to become a viable adjunct or alternative in ETW prevention.

### Acknowledgements

It is worth noting that the present *invitro* study was conducted as part of a PhD thesis. The authors would like to express their gratitude to Spyros Zinelis, Professor of Dental Materials, Department of Biomaterials (Head Prof. G. Eliades), School of Dentistry, NKUA, Greece, and all the members in the Department of Dental Materials for their assistance with analysis, interpretation, and for providing the necessary facilities and resources, which made the study possible.

The study was performed in accordance with the Declaration of Helsinki (WMA 2013) ethical standards and the research protocol was submitted and approved by the Ethics Committee of the School of Dentistry, National & Kapodistrian University of Athens, Greece (489/20.01.2022).
